# Tin Whiskers’ Behavior under Stress Load and the Mitigation Method for Immersion Tin Surface Finish

**DOI:** 10.3390/ma14226817

**Published:** 2021-11-11

**Authors:** Nor Akmal Fadil, Siti Zahira Yusof, Tuty Asma Abu Bakar, Habibah Ghazali, Muhamad Azizi Mat Yajid, Saliza Azlina Osman, Ali Ourdjini

**Affiliations:** 1 Department of Materials, Manufacturing, and Industrial Engineering, School of Mechanical Engineering, Faculty of Engineering, Universiti Teknologi Malaysia, Johor Bahru 81310, Malaysia; tuty@utm.my (T.A.A.B.); habibahghazali@utm.my (H.G.); azizi@utm.my (M.A.M.Y.); 2 Department of Mechanical and Manufacturing Technology, Kolej Vokasional Sungai Buloh, Jalan Kuala Selangor, U20, Shah Alam 40160, Malaysia; sitizahira@kvsbkpm.edu.my; 3 Faculty of Mechanical and Manufacturing Engineering, Universiti Tun Hussein Onn Malaysia, Parit Raja 86400, Malaysia; salizaz@uthm.edu.my; 4 Department of Mechanical Engineering, Faculty of Engineering, University of Ottawa, Ottawa, ON K1N 6N5, Canada; aourdjin@uottawa.com

**Keywords:** immersion tin coating, tin whiskers, micro-hardness indentation, copper substrate, JEDEC Standard JESD22-A121A, nickel underlayer

## Abstract

Since the use of the most stable Pb-based materials in the electronic industry has been banned due to human health concerns, numerous research studies have focused on Pb-free materials such as pure tin and its alloys for electronic applications. Pure tin, however, suffers from tin whiskers’ formation, which tends to endanger the efficiency of electronic circuits, and even worse, may cause short circuits to the electronic components. This research aims to investigate the effects of stress on tin whiskers’ formation and growth and the mitigation method for the immersion of the tin surface’s finish deposited on a copper substrate. The coated surface was subjected to external stress by micro-hardness indenters with a 2N load in order to simulate external stress applied to the coating layer, prior to storage in the humidity chamber with environmental conditions of 30 °C/60% RH up to 52 weeks. A nickel underlayer was deposited between the tin surface finish and copper substrate to mitigate the formation and growth of tin whiskers. FESEM was used to observe the whiskers and EDX was used for measuring the chemical composition of the surface finish, tin whiskers, and oxides formed after a certain period of storage. An image analyzer was used to measure the whiskers’ length using the JEDEC Standard (JESD22-A121A). The results showed that the tin whiskers increased directly proportional to the storage time, and they formed and grew longer on the thicker tin coating (2.3 μm) than the thin coating (1.5 μm). This is due to greater internal stress being generated by the thicker intermetallic compounds identified as the Cu_5_Sn_6_ phase, formed on a thicker tin coating. In addition, the formation and growth of CuO flowers on the 1.5 μm-thick tin coating suppressed the growth of tin whiskers. However, the addition of external stress by an indentation on the tin coating surface showed that the tin whiskers’ growth discontinued after week 4 in the indented area. Instead, the whiskers that formed were greater and longer at a distance farther from the indented area due to Sn atom migration from a high stress concentration to a lower stress concentration. Nonetheless, the length of the whisker for the indented surface was shorter than the non-indented surface because the whiskers’ growth was suppressed by the formation of CuO flowers. On the other hand, a nickel underlayer successfully mitigated the formation of tin whiskers upon the immersion of a tin surface finish.

## 1. Introduction

The urge to use lead (Pb) or lead-based alloy-free materials in electronic packaging has become a serious problem among manufacturers since the European Union RoHS (Restriction of Hazardous Substances) regulated the minimum content of lead in products starting from 1 July 2006 [1,2,3]. Therefore, countless research has been devoted in the past two decades to replace lead in solder and surface finish materials for electronic packaging.

The most critical design for the printed circuit board (PCB) structure is the surface finish deposited on the bare copper surface. Common surface finish deposition methods are electroless nickel/electroless palladium/immersion gold (ENEPIG) [4], electroless nickel/immersion gold (ENIG) [5], immersion silver (IAg) [6], immersion tin (ISn) [7], and the method most widely used by the industry is the hot air soldered levelled (HASL) method [8]. Even though HASL is the most popular method, it still uses lead in a significantly large portion [9]. To completely remove lead from the surface finish, pure tin plating has become the most popular option due to its good solderability and lower cost [10,11,12].

However, a major problem arises when the pure tin surface finish is used. The problem is mainly due to the formation of tin whiskers that potentially cause a short circuit if they come into contact [13,14,15,16]. Capacitors, diodes, and transistors with tin finishes are the most common electronic components that suffer from tin whiskers’ formation as discovered in the nuclear power plant [16], automotive [17] digital communication [18], and air force infrastructure [19] fields. An incident related to the shutdown of a nuclear reactor in 2005 revealed that tin whiskers were found on one of their PCB surfaces and were connected to each other, which created an electrical shortage to the components [20]. In another case related to the automotive industry in 2009, a car manufactured by Toyota was reported to have a problem on its pedal assembly [21]. The investigation conducted on the accelerator pedal position sensors (APP) of the car by Sood et al., 2011, revealed that the formation and growth of tin whiskers were actually the main cause for the electrical short circuit of the APP board [22].

The combination of certain factors such as an external force or stress, the interface morphology, temperature and humidity, residual stress, oxidized layer, and intermetallic compound (IMC) formation may influence the formation of tin whiskers. For example, the different morphologies and surface roughness of a Cu substrate can influence the behavior of tin whiskers’ formation and growth [23]. In another study, Gedney et al. found, on the other hand, that the behavior of IMC growth resulted in compressible stress being generated, thus promoting the whiskers’ growth [24]. The most common IMCs found were Cu_6_Sn_5_ and Cu_3_Sn, which form within the tin layer and the Cu substrate [9,24,25,26]. A detailed characterization of the Cu_6_Sn_5_ phase formation during heat treatment was reported by Johan et al. using 3DXRD [27]. Therefore, it is clearly understood that the tin layer itself can become a site for tin whiskers’ formation and growth to relieve further the stress. Possible mechanisms for tin whiskers’ formation and growth, such as dislocation, recrystallization, grain boundary diffusion, and interface fluid flow, have been proposed by researchers [24]. However, there is still uncertainty concerning the exact mechanism of tin whiskers’ formation due to the multiple factors that affect its behavior.

Several efforts have been devoted to mitigating the formation and growth of tin whiskers, such as reflow, post baking, annealing, conformal baking, and the most expensive method, metallic underlayer coatings [28,29,30]. On top of that, the reduction in metallic interdiffusion between the Cu lead frame and the surface finish has shown a promising outcome for mitigating the formation of tin whiskers [31].

Since all the electronic industries have moved towards lead-free electronic products, the most preferred alternative materials to Sn-Pb alloy are pure tin and tin-based alloys, and this is where tin whiskers become a major problem to the electronic packaging system. The idea that IMC formation generates internal compressive stresses at the interface between the tin layer and Cu substrate, thus resulting in whiskers’ growth out of the tin surface, which is a stress-free region, has motivated Moriuchi et al. to create a method to investigate tin whiskers’ behavior using external compressive stress [32]. They found that the mechanism of tin whiskers’ formation on a binary alloy of an Ag-Sn surface finish is due to the phase changes of β-Sn to α-Sn when an external force is loaded on the surface finish. This is one of the generation mechanisms of whiskers when a limited solid solubility alloy system consisting of two or more solid phases at room temperature is used for the surface finish.

Due to the potential of solid phase changes in the binary alloy system, among the lead-free surface metallurgies, pure tin plating has received greater attention in the industry owing to its single-phase at room temperature despite the excellent solderability and availability. The deposition of a tin surface finish using the electroplating process has been widely used because it is less expensive compared to the high-performance coatings such as chemical vapor deposition (CVD) and physical vapor deposition (PVD) [33]. Nonetheless, the recent development of a compact electronic packaging system showed the drawback of the electroplating process, where the minimum thickness is limited to 7 µm. The immersion process, however, has outperformed the electroplating process due to its capability to achieve a coating thickness as thin as 0.3 µm. Many of the previous studies on whiskers’ behavior have focused on the electroplating process of the tin surface finish and almost none have used the immersion process.

Therefore, the aim of this study is mainly to investigate and understand the tin whiskers’ behavior for the immersion tin surface finish by applying external stress through indentation, as well as the effectiveness of this method to mitigate whiskers’ formation and growth with a nickel underlayer.

## 2. Materials and Methods

The copper substrate is a print circuit board uniformly coated with a 20 µm copper layer supplied by GG Circuits Industries Sdn. Bhd., (Johor Bahru, Malaysia). The tin plating was deposited using the immersion process on the copper substrates. The copper substrates were pre-treated before the tin plating as follows:

1. Grinding with silicon carbide paper (800 grit) and rinsing with distilled water to remove the oxide layer.
2. Immersing in 2-Butoxyethanol solution at 70–90 °C for 5 min and rinsing with distilled water to remove the dirt.
3. Soaking in a NaOH solution at 60 °C for 3 min and rinsing with distilled water for surface degreasing.
4. Etching in a 10% H_2_SO_4_ solution for 1 min and rinsing with distilled water to deoxidize the surface.
5. Immersing in palladium chloride solution for 3 min and rinsing with distilled water to activate the surface.

Table 1 shows the chemical composition for tin plating [34]. The plating durations were 12 and 20 min to produce a coating thickness less than 2 µm and more than 2 µm, respectively. The temperature used during the plating process was 75 ± 1 °C controlled by a plating bath. Chen et al. [9] stated that after a deposition time of 10 min, the thickness of the tin coating is about 0.7–2 µm, which is considered to be a sufficient thickness to promote whiskers’ formation.

For the tin whiskers’ mitigation experiment, a nickel underlayer was deposited prior to the tin plating layer using the electroless plating process. In order to test the effectiveness of Ni underlayers in mitigating the whiskers’ formation, two different nickel coating thicknesses were deposited under the 1.5 µm immersion tin coating thickness. The plating times were 10 and 20 min to produce a uniform layer of the nickel coating with thicknesses of 2.4 and 2.7 µm, respectively. The chemical composition and parameters of the electroless nickel plating are shown in Table 2 [7,35].

The storage test was conducted at a constant temperature and humidity in the humidity chamber (MEMMERT HCP 108, manufactured by Memmert GmbH, Schwabach, Germany). The temperature and humidity were controlled at a constant value of 30 °C/60% RH following the JEDEC Standard JESD22-A121A for testing the whiskers’ growth [36]. The samples were stored in the humidity chamber right after the plating process was completed. For each parameter studied, the storage test was carried out exactly the same on the same samples with the time intervals of 1, 4, 8, 12, and 52 weeks. The characterization and whisker’s length measurements were carried out on the sample at the specified time interval before being restored in the humidity chamber. There were eight samples prepared in this research as shown in Table 3.

Many researchers [32,37,38,39] have reported that indentations can act as a source of external stress that accelerates the whiskers’ growth, but none have focused on the immersion tin surface finish in detail. In this research, the indentation was conducted using the HMV SHIMADZU (Shimadzu Corporation, Nakagyo, Kyoto, Japan) micro-hardness tester to induce the external stress on the tin coating. The indentation test was applied on the fresh plated samples to study the whiskers’ growth inside the indentation and nearby areas [25]. A 2N load was used in this study to generate the stress on the surface for the coating thickness less than 9 µm [32]. The 2N load was used to ensure that the indenter would penetrate through the coating to the Cu substrate. The penetration depth of the indenter into the Cu substrate was 60% or larger from the total depth of penetration of the micro-hardness indenter. Therefore, the effect of mechanical properties on different thicknesses of the surface finish could be negligible. The indented samples were stored under temperature/humidity conditions as stated above in the humidity chamber to investigate the effects of environmental conditions on whiskers’ growth.

The as-plated surface finish was characterized by measuring the thickness of the cross-sectional samples using an optical microscope equipped with an image analyzer (Nikon-Eclipse LV150 by Nikon Corporation, Shinagawa, Tokyo, Japan) and i-Solution Lite software. The tin whiskers’ behavior and IMCs were observed using field emission scanning electron microscopy (FESEM (Supra-35VP, by Carl Zeiss, Oberkochen, Germany) and the elemental analysis was performed by an energy dispersive X-ray (EDX). The measurement of whiskers’ lengths was performed by observation at the top view of the FESEM micrograph using i-Solution Lite software following the JEDEC standard No. 22-A121A [36]. Basically, the length of the tin whiskers was determined based on the average length of the five longest whiskers per sample.

The top-view IMC observation by FESEM and elemental analysis by EDX was performed on the Cu–Sn interface to verify the existence of IMCs between the copper substrates and tin that developed due to the chemical reaction that occurs during the plating process. The as-plated tin surface finish was etched in the etchant solution of nitric acid (40 g), sulfamic acid (2 mL), fluoroboric acid (1.5 mL), and distilled water (60 g) for 5 min.

## 3. Results and Discussion

### 3.1. Characterization of Immersion Tin Coating

**Figure 1:**
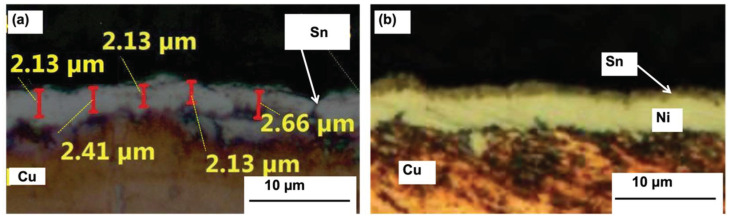
Cross-sectional images of (**a**) tin and (**b**) Ni underlayers deposited on copper substrate.

Figure 1 shows the cross-sectional image of (a) a tin coating (plating time = 20 min) and (b) a tin/Ni underlayers coating (plating time; nickel = 20 min, tin = 12 min). The results show that tin and Ni were dense and uniformly coated with the Cu substrate. The coating thickness of the immersion tin surface finish was measured by an image analyzer, and the thickness has been confirmed as 1.5 and 2.3 µm for 12 and 20 min plating durations, respectively. The Ni coating thickness has been confirmed as 2.4 and 2.7 µm for 10 and 20 min plating durations, respectively.

**Figure 2:**
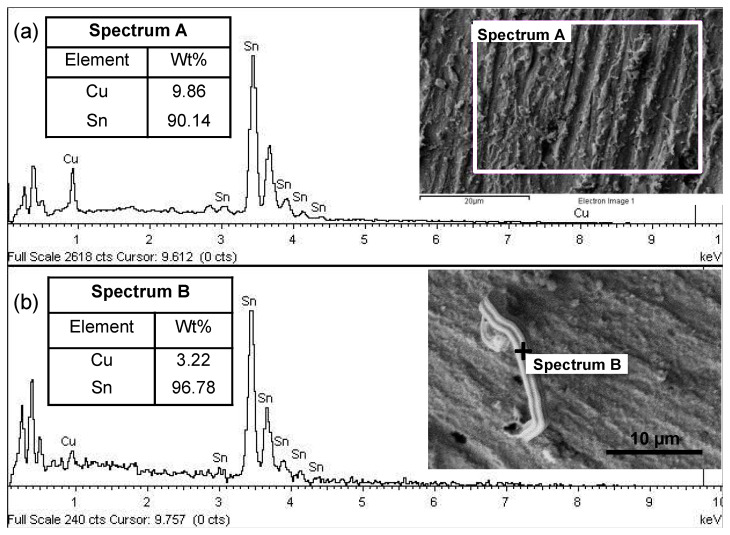
EDX spectra of the selected area in the FESEM micrograph of the immersion tin surface finish (**a**) and point analysis on selected tin whiskers’ surface (**b**). The inserted table shows the chemical composition of the measured surfaces. The inserted image is the FESEM micrograph of the measured surfaces.

The chemical composition analysis of the tin-coated substrate was performed using EDX equipped with FESEM on a freshly plated sample to confirm that pure Sn has been deposited on the Cu substrate. Figure 2a shows the EDX spectra of the selected area on the sample with a 12 min plating duration (1.5 µm tin coating thickness), the FESEM micrograph of the scanned area, and the chemical composition results as in the table. Spectrum A is the marked area on the immersion tin surface, which is measured by EDX. Based on the weight percentage (wt.%) of elements presented in the table, it was clearly shown that only pure Sn was deposited on the Cu substrate with 90.14 wt.%, and a small amount of Cu was detected from the substrate itself.

The chemical composition of the whiskers was measured with EDX on the 1.5 µm tin coating after 1 week in storage. Figure 2b shows the EDX spectra of the detected point marked as Spectrum B on the tin whisker. The inserted image is a FESEM micrograph of the whiskers sample and the chemical composition results are shown in the inserted table in Figure 2b The results clearly show that 96.78 wt.% Sn was detected on the whisker. Therefore, the structure is confirmed as a pure tin whisker, which is consistent with previous work in the literature [32,40,41].

### 3.2. Whiskers’ Behavior on Non-Indented Tin Coating

**Figure 3:**
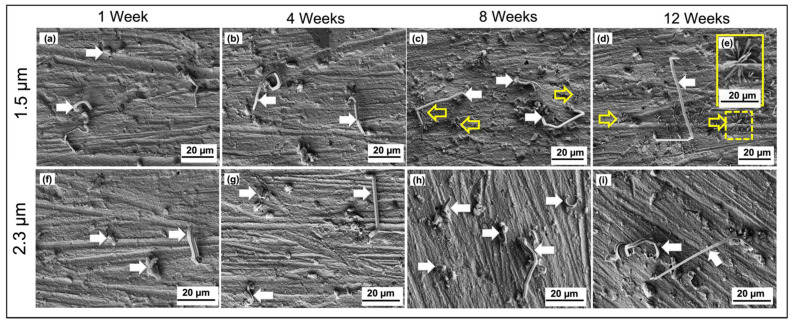
FESEM micrographs of tin whiskers (solid arrows) and CuO flowers (hollow arrows) observed on the non-indented surface of 1.5 µm (**a**–**e**) and 2.3 µm (**f**–**i**) coatings for 1–12 weeks of storage time. Image (**e**) is the high-magnification image of CuO flowers (hollow arrow) in the marked area in image (**d**).

The effect of external stress on the whiskers’ formation and growth was studied by observing the whiskers’ behavior for indented samples and comparing the results with the non-indented surface finish. The indentation was made using a micro-indenter to generate external stress on the tin surface. Figure 3a–e shows the FESEM micrographs of the tin whiskers (solid arrows) that formed on the non-indented surface of the 1.5 µm tin coating during 1–12 weeks of storage time. The results showed that the whiskers grew longer with longer storage time. as the shorter tin whiskers can be seen clearly in Figure 3a while longer tin whiskers are shown in Figure 3c,d. Several types of tin whiskers were found on the samples, such as straight, bent, spiral, kinked, and spiral, with striations along the circumference. The longest tin whiskers were observed in the sample after 12 weeks. The kinked-type whiskers were identified as the most dominant type of tin whiskers formed on the samples after 8 and 12 weeks. As explained by Donald Susan et al. [42], if the kinked-type whiskers did not grow in diameter but did in length, and its end-tip is facing the direction normal to the tin surface, it will continuously grow longer. In addition, after 12 weeks of storage in a controlled environment, it was found that CuO flowers formed on the tin surface as marked by hollow arrows in Figure 3d. The enlarged image of CuO flowers is shown in Figure 3e inside Figure 3d.

A similar trend has been shown by the non-indented surface of the 2.3 µm tin coating as shown in Figure 3f–i. However, the length of the tin whiskers on the thicker coating of the immersion tin surface finish (2.3 µm) is longer than the length of the tin whiskers on the thin coating. As expected, the thickness of the tin coating influenced the whiskers’ formation and growth. This can be explained by the excessive amount of Sn atoms in the thicker tin coating that caused sufficient Sn supplies to form longer whiskers as compared to the thinner tin coating, which had fewer Sn atoms.

**Figure 4:**
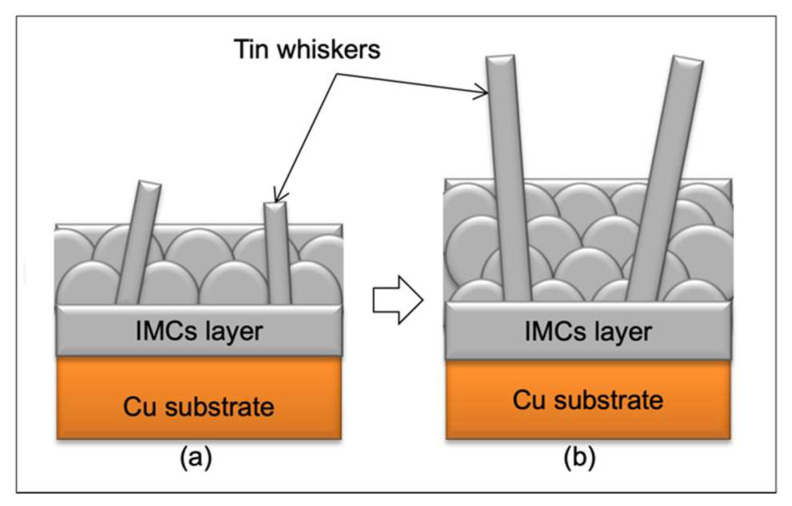
A schematic diagram of the whiskers’ formation on thin (**a**) and thick (**b**) tin coatings of immersion surface finish.

Figure 4 shows a schematic diagram that demonstrates the whiskers’ formation on thinner and thicker tin coatings. The results were in agreement with previous studies [33,34] as they reported that longer whiskers on the electroplated tin coating were found at a tin thickness of 2 µm as compared to 1 µm. The mechanism of the whiskers’ growth has been well discussed in the literature [11,35], where the growth has been understood to occur at the base of the whiskers and not at the tip, since the tip remains unchanged after the whiskers have grown longer. This is consistent with the IMC theory, which states the formation of whiskers is due to the stress generated within the tin coating and the whiskers grow longer when the IMCs have grown thicker over time.

### 3.3. Whiskers’ Behavior on Indented Tin Coating

**Figure 5:**
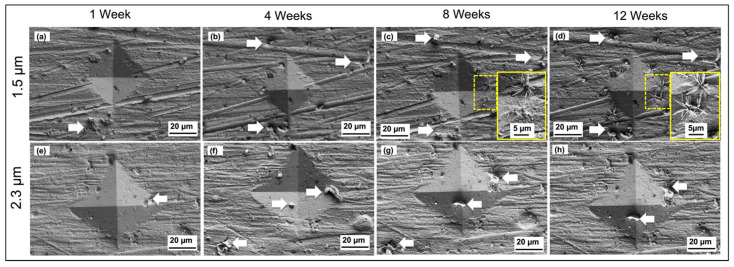
FESEM micrographs of tin whiskers observed on the indented surface of 1.5 µm (**a**–**d**) and 2.3 µm (**e**–**h**) tin coatings after 1 week up to 12 weeks of storage time.

For the indented samples, Figure 5 shows the FESEM micrographs of the whiskers in the indentation area and stress-affected area around the indented point for week 1 through to week 12 for 1.5 and 2.3 µm tin coatings. For the 1.5 µm tin coating, (Figure 5a–d), three solid arrows point out exactly the same tin whiskers formed on the coating and copper oxide, while the CuO flower structure is highlighted in yellow boxes in Figure 5c,d. From the results, no tin whiskers were found inside the indentation point except at the edge and outside the indentation point after 1 week of storage (Figure 5a), and the whiskers increased in length at week 4 (Figure 5b) and remained constant after week 4 (Figure 5c,d). Moreover, more tin whiskers were found in the region outside the indented area.

The tin whiskers observed on the 2.3 µm tin coating in Figure 5e–h showed behavior consistent with the whiskers formed on the 1.5 µm tin coating. The results also showed consistent behavior of the tin whiskers formed on the 1.2 µm tin coating of the immersion tin surface finish as reported in previous research [36]. In general, the number of whiskers formed inside the indentation area is less than the ones outside the dented region for all of the coating thicknesses. This is because the outer region has a lower stress concentration as compared to the inside of the indentation area. The diamond shape of the indented sample has higher stress concentrated at the center tip of the diamond shape and the stress decreases towards the outer region of the diamond shape. Thus, this can be explained by the migration of Sn atoms from a higher- to a lower-stress region to release the stress [38].

The discontinuity of the whisker’s growth is related to the creep process that occurred in the tin surface and the formation of copper oxide, CuO. The creep process in the tin plating contributes to the relaxation of the external stress generated by the micro-indentation under the storage temperature, thus it causes discontinued growth of whiskers in the indented surface. Since the melting point, T_m_, of pure tin is 263 °C (536 K) and the creep temperature is 0.4 T_m_ (214.4 K), at the storage temperature of 30 °C (303 K), creep therefore dominated because it is above creep temperature. In short, the stress generated by the indentation was gradually released during the creep process. Another reason for the discontinuity of tin whiskers’ growth at week 8 and onwards is the formation of CuO flowers. Based on Figure 5c, a large number of CuO flowers was observed inside the indentation area and its surrounding areas for the samples stored under 30 °C/60% RH for 8 weeks and 12 weeks. Further investigation has been conducted on the CuO flower formation since it is believed to be one of the factors contributing to the discontinuity of whiskers’ growth.

Based on Figure 5a, whiskers formed as early as week 1 and continued to grow up to week 4 without the existence of CuO flowers. However, CuO flowers were observed inside the indentation areas and its surrounding areas at week 8 and they had increased in number and size by week 12 while the whiskers had not grown longer by week 8 and week 12. Thus, the results proved that the formation of CuO flowers contributed to the discontinuity of whiskers growth because of the stress released during the formation of CuO flowers. CuO flowers have been forced to form on the tin coating surface due to the stress generated from the indentation. The indentation forced a fast reaction between the copper substrate and the tin film coating. Therefore, the diffusion of copper penetrated into the very thin film of the tin coating to react with the oxygen to form CuO flowers. This is true as the number of CuO flowers was greater on the thinner tin coating (1.5 µm) shown in Figure 5a–d as compared to the thicker tin coating (2.3 µm) shown in Figure 5e–h. The formation of CuO flowers after week 4 suppressed the whiskers’ growth. The oxidation activity of Cu from the substrate took place during the Sn atoms’ migration to form CuO flowers instead of forming tin whiskers or extending the length of the whiskers.

**Figure 6:**
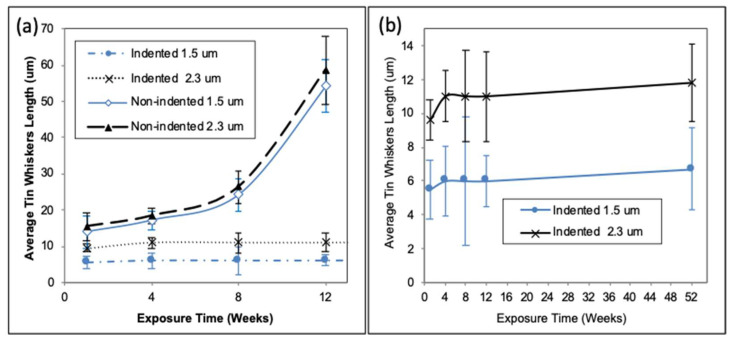
(**a**) The average tin whisker length versus storage times for non-indented and indented surfaces after being stored up to 12 weeks. (**b**) The tin whiskers’ length at the indentation points over storage time up to 52 weeks.

A comparison study on whiskers’ length was conducted by measuring the whiskers’ length on the non-indented surface as well as the whiskers’ length formed inside the indentation area for indented samples as shown in Figure 6a. According to the graph, for the non-indented samples, the whiskers’ length grew over time after 12-week storage under 30 °C/60% RH conditions. The growth of whiskers was influenced by the IMC formation. The IMCs formed as the tin coating was deposited on the copper substrate and grew further over the storage time. The formation of IMCs generates stress within the tin coating, thus resulting in the diffusion of Sn atoms towards the stress-free zone to form whiskers. Hence, further growth of IMCs over time caused longer whiskers to form due to the higher stress generated by IMCs. These results were in agreement with previous studies that proved that tin whiskers were grown over longer storage times as a result of the continuous formation of IMCs, which is Cu_6_Sn_5_ [37,38,39,40]. However, in the case of the indented surface, the whiskers’ behavior showed a different trend. The whisker length on the 1.5 µm tin coating at week 1 was 5.6 µm and the length slightly increased at week 4 (6.0 µm) and remained constant after week 4 until week 12. The whisker length on the 2.3 µm tin coating slightly increased from week 1 (thickness = 9.6 µm) to week 4 (thickness = 11 µm) and then remained constant up to week 12. From the results, it is clearly shown that the whiskers’ growth on the indented surface was discontinued even after 4-week storage under 30 °C/60% RH. Even after 52 weeks of storage, the whiskers at the indentation point showed a very small increment as shown by the graph in Figure 6b. Both 1.5 and 2.3 µm tin coatings showed a slight increment in whisker length after 52 weeks of storage with lengths of 6.7 and 11.8 µm, respectively.

### 3.4. Whiskers Behavior as a Function of Load Distance

In a previous discussion, it was concluded that there is no significant increase in whisker length after 4 weeks stored under 30 °C/60% RH up to 12 weeks for an indented surface. The initial whisker analysis was focused on the indentation point only for a comparison study between the indented and non-indented surface. A further investigation was conducted to study the whiskers’ behavior at the area farther from the indentation point, which is known as the stress-affected area. The whisker analysis at a specific distance from the indentation point was conducted on the sample after 52 weeks stored under 30 °C/60% RH conditions.

**Figure 7:**
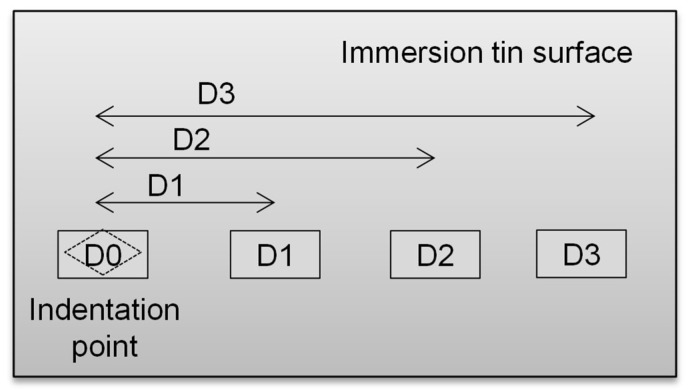
A schematic diagram to indicate the position on the immersion tin surface to be observed by FESEM.

Figure 7 shows the schematic diagram to indicate the position on the immersion tin surface to be observed by FESEM after 52 weeks of storage under 30 °C/60% RH. Position D0 is the indentation point, while positions D1, D2, and D3 are the positions at a certain distance (D1, D2, and D3, respectively) from the indentation point, which were selected randomly to indicate the changes in the tin whiskers’ behavior at different positions farther from the indentation point.

**Figure 8:**
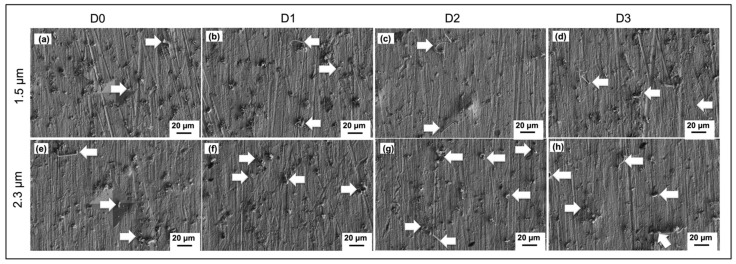
FESEM micrograph on the whiskers’ formation after 52 weeks stored under 30 °C/60% RH at a certain distance from the indented point for 1.5 µm (**a**–**d**) and 2.3 µm (**e**–**h**) coatings of the immersion tin surface finish (refer to Figure 7 for regions D0, D1, D2, and D3).

Figure 8 shows the FESEM micrographs on whisker formation after 52 weeks of storage time at a certain distance from indented points on the 1.5 and 2.3 µm tin coatings, respectively. Based on Figure 8, the number of whiskers formed is greater and the length of whiskers is longer at the position farthest from the indentation point. Besides that, the number of CuO formed on the surface is greater at a position close to the indentation point and gradually decreased at the position farthest from the indentation point. This is in agreement with the fact that the CuO flower influenced the growth of the tin whiskers. However, a greater CuO flower can be observed on the thinner tin coating, 1.5 µm (Figure 8a–d), as compared to the thicker tin coating, 2.3 µm (Figure 8e–h).

**Figure 9:**
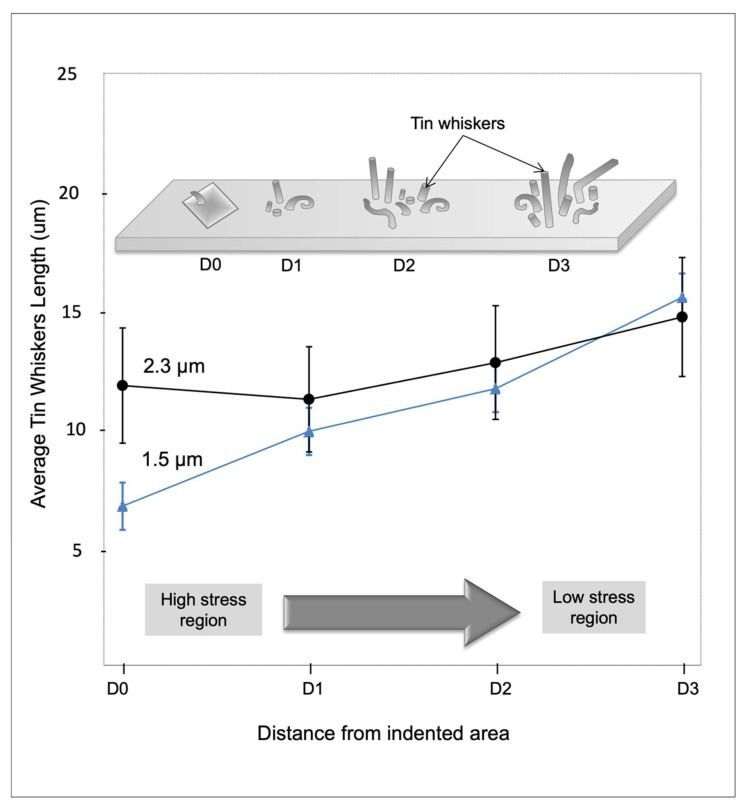
The average whisker length over the distance from the indentation point after 52 weeks stored under 30 °C/60% RH for 1.5 and 2.3 µm tin coatings. Inserted figure: The schematic diagram of tin whiskers formed on the immersion tin surface finish at a specific distance from the indentation point.

Figure 9 shows the graph of the average whisker length at positions D0, D1, D2, and D3 after being stored for 52 weeks. Based on the graph, the whiskers’ length is directly proportional to the distance from the indentation point after 52 weeks stored under 30 °C/60% RH conditions for both 1.5 and 2.3 µm tin coatings. The average whisker length at the indentation point, D0, was 6.7 µm, while at D3, the farthest from the indentation point, it was 15.53 µm for the 1.5 µm tin coating. Meanwhile, for the thicker coating, 2.3 µm, the average whisker length was 11.8 µm and 14.7 µm for locations D0 and D3, respectively. The results have proved that the whiskers tend to form at a lower stress region as longer whiskers formed at the farther distance, D3, from the indentation point, D0. This is because Sn atoms move from a high-stress region to a lower-stress region to release stress within the tin coating as critically explained by N. Muraki and G Katagiri [43]. As mentioned previously, the indentation point has the highest stress concentration, and the stress decreases at a farther distance. However, the maximum average tin whisker length at the farthest distance from the indentation point, D3, is still far shorter than the non-indented surface, which is only one quarter of the average length. The normal IMC growth generated stress, thus pushing the Sn atoms to migrate and form tin whiskers at the surface to release the stress. However, by applying external stress, where the stress amount is much greater than the natural stress generated by IMC growth, CuO was influenced to form on the surface faster than the formation of the tin whiskers. The major stress generated from the indentation was released by the formation and growth of the CuO flowers.

### 3.5. Influence of Inter-Diffusion

To study the influence of inter-diffusion at the interface of the tin coating and the Cu substrate, a Ni underlayer was deposited between the Cu substrate and the tin coating to mitigate the tin whiskers’ formation on the tin surface finish. Different thicknesses of the Ni underlayer, 2.4 and 2.7 µm, were deposited under the 1.5 µm tin coating, and the whisker analysis was conducted on the 12th week of storage under 30 °C/60% RH conditions.

**Figure 10:**
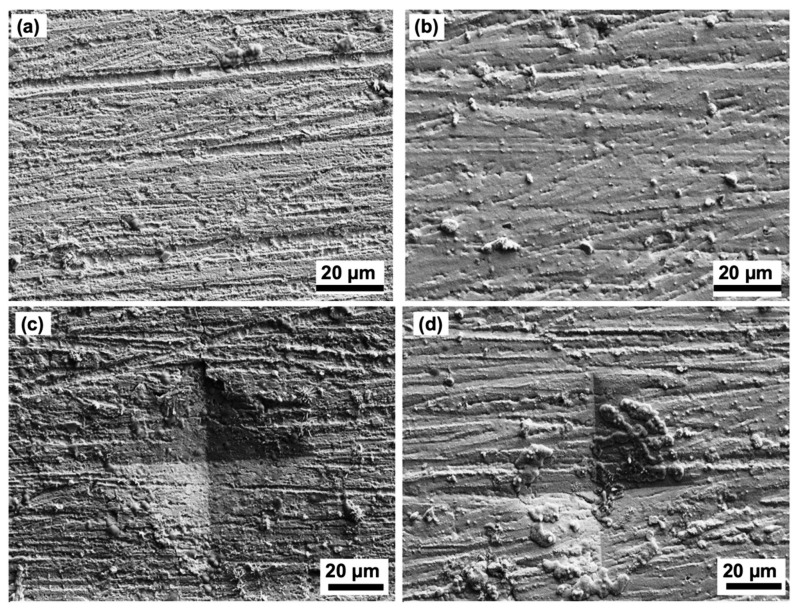
FESEM micrographs of the non-indented and indented surfaces after 12-week storage at 30 °C/60% RH with various coatings of Sn/Ni layer: (**a**) Non-indented, Sn/Ni = 1.5/2.4 µm, (**b**) non-indented, Sn/Ni = 1.5/2.7 µm, (**c**) indented, Sn/Ni = 1.5/2.4 µm, and (**d**) indented, Sn/Ni = 1.5/2.7 µm.

Figure 10a,b shows the FESEM micrographs of the non-indented Sn/Ni surfaces for Sn/Ni thicknesses of 1.5/2.4 and 1.5/2.7, respectively, after 12 weeks of storage at 30 °C/60% RH. According to Figure 10a,b, no whiskers were observed on the tin surface finish after 12 weeks of storage. Similar results were shown by the FESEM micrographs on the indented surface in Figure 10c,d where no whiskers were observed on the indented surface for Sn/Ni thicknesses of 1.5/2.4 and 1.5/2.7, respectively, after 12 weeks stored at 30 °C/60% RH. Therefore, both thicknesses of the Ni underlayer were effective to mitigate the formation of whiskers, even through the external stress generated on the tin surface.

Since the Ni underlayer can mitigate the formation of the whiskers for up to 12 weeks in this study, the results are consistent with previous works for the electroplated tin surface finish as it was proposed that the Ni underlayer can delay the whiskers’ formation. Ho et al. [44] mentioned that the growth rate of the Cu_3_Sn can be reduced with the presence of a Ni underlayer. Similar findings were also shown by a study conducted by Hashim et al. on the whisker mitigation method using a Ni underlayer, but they used a different tin plating, which was dipped in a molten Sn bath [45]. According to Laurila et al. [46], the reaction rate of the Ni–Sn is relatively smaller than the Cu-Sn, thus resulting in a thinner IMC layer in the Ni–Sn. Other researchers also agreed that the reaction rate of Ni–Sn is slower compared to the reaction rate of the Cu-Sn [47,48]. Thus, the Ni layer acts effectively as a barrier layer for the immersion tin surface finish to prevent the formation of Cu–Sn intermetallics, which is believed to be the main factor for whiskers’ formation and growth.

**Figure 11:**
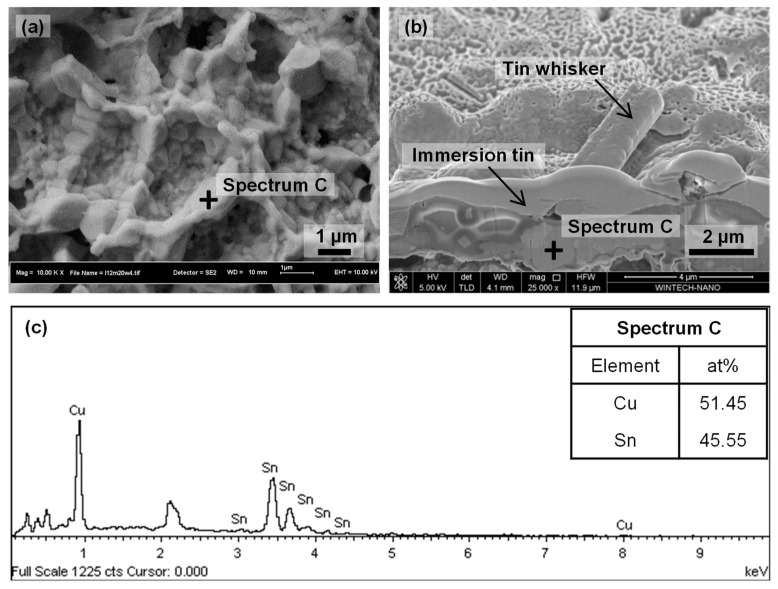
FESEM micrographs of IMCs on 1.5 µm tin coating from (**a**) top and (**b**) cross-sectional views after 20 weeks of storage at 30 °C/60% RH. The EDX spectra of IMCs is shown in (**c**) with elemental composition in the table inserted in (**c**).

An intermetallic compounds (IMCs) analysis was conducted on selected samples. The IMC formation for the 1.5 µm tin coating after 20 weeks of storage at 30 °C/60% RH was studied from the top (Figure 11a) and cross-sectional (Figure 11b) views. The cross-sectional view of IMCs was performed by a focused ion beam (FIB) equipped on the FESEM. The chemical composition of the IMCs was determined by EDX and the results are shown in Figure 11c. From the top-surface observation shown by the FESEM micrograph in Figure 11a, it can clearly be seen that the spherical shape of IMC formed at the interface between the Cu substrate and tin coating and was greater along the grain boundary of the immersion tin grain. This is true as the grain boundary is typically a weak spot and easy target for chemical diffusion due to a high density of atom vacancies. Moreover, the IMCs covered the entire surface of the copper substrate. The EDX analysis confirmed that the IMC (marked as Spectrum C in Figure 11a,b) is Cu_6_Sn_5_ as calculated based on the 54.45 at.% Cu and 45.55 at.% Sn composition shown by the EDX results in Figure 11c. The thermodynamics calculation method of the Cu-Sn binary system for determining the IMC based on the atomic percentage of elements has been discussed by Wojcieh Gierlotka et al. [45]. According to a cross sectional image in Figure 11b, the IMC layer formed between the tin whiskers and copper substrate. Since the results showed that the tin whiskers formed above the IMC layer, the theory regarding IMCs generating compressive stress within the Sn–Cu film and causing tin whiskers’ formation is proven.

The samples with a Ni underlayer showed no signs of tin whiskers’ formation due to the formation of stable IMCs of Ni_3_P uniformly at the interface with no trace of Cu on the surface, as explained in the literature [49,50]. All the mentioned studies have confirmed that there were no Cu-Sn IMCs found in the Ni underlayer samples. It has been proven that the Ni underlayer can completely prevent the fast diffusion of Cu-Sn to form Cu-Sn IMCs, thus preventing the whiskers’ formation.

## 4. Conclusions

A study on external stress and Ni underlayer effects on tin whiskers’ behavior for the immersion tin surface finish was conducted. The samples were stored under 30 °C/60% RH environmental conditions for a period of up to 52 weeks and were monitored at weeks 1, 4, 8, 12, and 52. Based on the surface morphology and intermetallic (IMC) analysis, the conclusions are as follows:

- Increasing the tin plating thickness up to 2.3 µm causes an increase in the tin whiskers’ length.
- The tin whiskers’ length is increased directly proportional to the storage time.
- As for the effects of externally applied stress, tin whiskers formed inside and outside the indented area except for the center tip of the indented area, since the tip has a high stress concentration while the other parts of the area have a lower stress concentration. The whiskers’ length increased with the increasing distance from the indented area.
- The formation and growth of the CuO flowers on the indented surface suppressed the growth of the tin whiskers.
- The whiskers formed as various types, including straight, bent, kinked, and spiral with striations along its circumference.
- The Ni underlayer acted as a barrier layer, which effectively mitigated the formation of tin whiskers since no whiskers were observed on the tin surfaces up to week 12 by slowing down the inter-diffusion between the Cu and Sn atoms.
- The identified IMC that influenced the formation and growth of the tin whiskers that formed at the interface between the immersion tin surface finish and the Cu substrate was Cu_5_Sn_6_.

## 5. Recommendation for Future Work

This study has proven that external stress generated by indentation can promote the formation and growth of tin whiskers, and the whiskers were successfully mitigated by a Ni underlayer deposited between the immersion tin surface finish and the Cu substrate, consistent with other coating methods such as electroplating [44] and hot-dip [45]. It will be interesting to further investigate the effect of stress distribution and residual stress to further understand the effect of external stress on the tin whiskers’ formation and behavior. Therefore, the authors would suggest a quantitative analysis on the stress distribution using the nano-indentation method on various tin plating methods such as immersion, electroplating, and hot-dip methods. Measuring the exact stress distribution is important to develop a stress-relief method for use in the manufacturing process of electronic packaging.

## Figures and Tables

**Table 1:** Chemical composition for tin plating [34].

Chemical	Function	Composition
Stannous chloride	Source of Sn ion	20 g/L
Hydrochloric acid (37%)	Base bath	37 mL/L
Sulfuric acid (50%)	Base bath	50 mL/L
Sodium hypophosphite	Reducing agent	16 g/L
Thiourea	Complexing agent	200 g/L
Phenolsulfonic acid	Bath stabilizer	5 mL/L
Temperature		75 ± 1 °C
Duration		12, 20 min

**Table 2:** Chemical composition and parameters of electroless nickel plating [7,35].

Chemical	Function	Quantity
Nickel sulphate	Source of Ni ion	28 g/L
Sodium acetate	Complexing agent	17 g/L
Sodium hypophosphite	Reducing agent	24 g/L
Lead acetate	Bath stabilizer	0.0015 g/L
Parameters		
pH		4.4–4.6
Temperature		85 ± 1 °C
Time		10 and 20 min

**Table 3:** List of samples.

No.	Coating	Plating Time (min)	Coating Thickness (µm)	Indentation	Storage Time (Weeks)
1	Sn	2	1.5	No	2, 4, 8, and 12
2	Sn	20	2.3	No	2, 4, 8, and 12
3	Sn	12	1.5	Yes	2, 4, 8, 12, and 52
4	Sn	20	2.3	Yes	2, 4, 8, 12, and 52
5	Sn/Ni	12/10	1.5/2.4	No	2, 4, 8, and 12
6	Sn/Ni	12/20	1.5/2.7	No	2, 4, 8, and 12
7	Sn/Ni	12/10	1.5/2.4	Yes	2, 4, 8, and 12
8	Sn/Ni	12/20	1.5/2.7	Yes	2, 4, 8, and 12

## Data Availability

The data presented in this study are available on request from the corresponding author.
